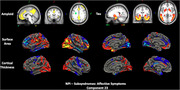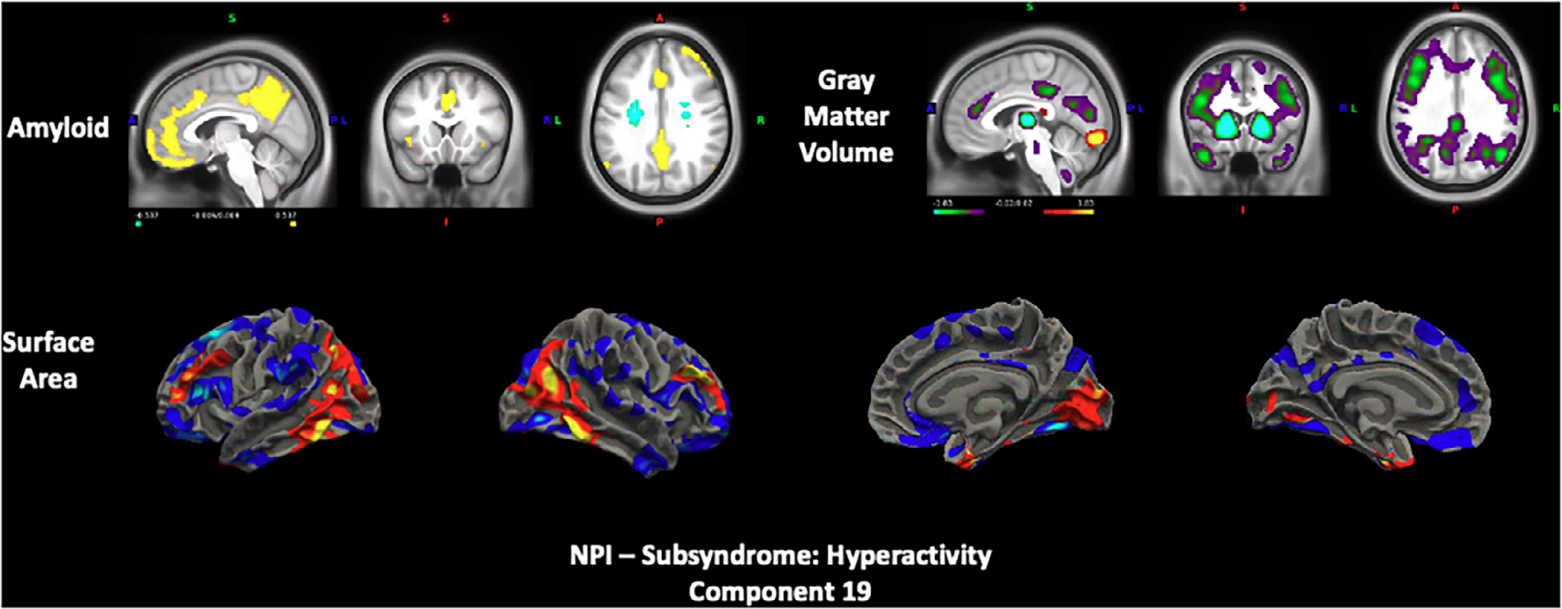# Multi‐Modal Neuroimaging Fusion Reveals Predictive Patterns of Affective Symptoms and Hyperactivity in Alzheimer’s Disease

**DOI:** 10.1002/alz70862_110135

**Published:** 2025-12-23

**Authors:** You Cheng, Adrián Medina, Cole Harris Korponay, David G. Harper, Lisa D Nickerson

**Affiliations:** ^1^ McLean Hospital, Belmont, MA USA; ^2^ McLean Hospital, Harvard Medical School, Belmont, MA USA

## Abstract

**Background:**

Neuropsychiatric symptoms (NPS) are prevalent in Alzheimer’s disease (AD) and may reflect distinct pathophysiological pathways from cognitive decline. While pathological and structural factors relate to NPS, the interplay of amyloid‐ tau‐neurodegeneration remains understudied. Using semi‐supervised learning, we identified multi‐modal neuroimaging patterns predictive of affective symptoms and hyperactivity.

**Method:**

Alzheimer’s Disease Neuroimaging Initiative (ADNI‐3) data included amyloid PET (A), tau PET (T), and structural MRI (N: cortical thickness, surface area, gray matter volume). Affective symptoms (anxiety/depression) and hyperactivity (agitation, irritability, euphoria, aberrant motor behavior, disinhibition) subsyndromes were derived from Neuropsychiatric Inventory (NPI) composite scores (frequency × severity). SuperBigFlica (SBF), a semi‐supervised fusion framework, decomposed modalities into shared latent components while jointly predicting affective /hyperactivity. SBF fused all five modalities, extracting 50 latent components mapped to A‐T‐N spatial patterns. Transfer learning validated biological relevance via ElasticNet regression predicting age and CDR‐SOB. Performance was assessed using Pearson correlation (95% confidence intervals [CI] bootstrapped 5000 iterations).

**Result:**

The cohort included 274 participants (192 training, 41 validation, 41 test; mean age 70.8 ± 6.9 years; 55.8% female; 92.7% White). Clinical measures included CDR‐SOB (median interquartile range [IQR]: 0 [0,1]) and NPI (median IQR: 0 [0,3]). The SBF model showed good predictive performance for affective symptoms (r=0.34, 95% confidence interval [CI]: 0.03–0.55) and hyperactivity (r=0.38, 95% CI: 0.06–0.72) in an independent test set. Affective symptoms were linked to default mode network (DMN) disruptions, with elevated amyloid (frontal‐parietal), elevated tau (middle temporal), and reduced cortical thickness and surface area (frontal‐temporal, temporal‐parietal), aligning with DMN’s role in emotional regulation (Figure 1). Hyperactivity correlated with increased amyloid and reduced gray matter and surface area in DMN and frontoparietal control network. Cingulate atrophy was associated with disinhibition and agitation, supporting its role in behavioral regulation (Figure 2). Transfer learning demonstrated generalizability, with SBF‐derived latent representations predicting age (r=0.45, 95% CI: 0.2–0.63) and CDR‐SOB (r=0.36, 95% CI: 0.05–0.61), validating the neuroimaging patterns’ biological relevance.

**Conclusion:**

This study identifies distinct A‐T‐N signatures for NPS subtyping in AD, with clinical relevance supported by predictive utility. Future work will validate these patterns longitudinally and across datasets.